# The Use of High-Concentration Collagen-Based Composition and Gelatin Granules as Bioinks for Extrusion 3D Bioprinting of Porous-Structured Hydrogel Constructs

**DOI:** 10.17691/stm2025.17.5.02

**Published:** 2025-10-31

**Authors:** A.A. Kisel, E.V. Isaeva, E.E. Beketov, N.V. Arguchinskaya, V.R. Gusarova, A.O. Yakimova, G.A. Demyashkin, T.S. Lagoda, D.S. Baranovsky, I.D. Klabukov, S.A. Ivanov, P.V. Shegay, A.D. Kaprin

**Affiliations:** Researcher, Laboratory of Tissue Engineering; The A. Tsyb Medical Radiological Research Centre — the Branch of National Medical Research Radiological Centre of the Ministry of Health of the Russian Federation, 10 Zhukov St., Obninsk, 249036, Russia; PhD, Senior Researcher, Laboratory of Tissue Engineering; The A. Tsyb Medical Radiological Research Centre — the Branch of National Medical Research Radiological Centre of the Ministry of Health of the Russian Federation, 10 Zhukov St., Obninsk, 249036, Russia; PhD, Director of Development Center; Mabscale LLC, 5A Highway No.4, Special Industrial and Production Economic Zone, Tolyatti, 445043, Russia; Junior Researcher, Laboratory of Tissue Engineering; The A. Tsyb Medical Radiological Research Centre — the Branch of National Medical Research Radiological Centre of the Ministry of Health of the Russian Federation, 10 Zhukov St., Obninsk, 249036, Russia; Biologist, Laboratory of Post-Radiation Recovery, Department of Radiation Biochemistry; The A. Tsyb Medical Radiological Research Centre — the Branch of National Medical Research Radiological Centre of the Ministry of Health of the Russian Federation, 10 Zhukov St., Obninsk, 249036, Russia; PhD, Head of the Laboratory of Molecular and Cellular Radiobiology; The A. Tsyb Medical Radiological Research Centre — the Branch of National Medical Research Radiological Centre of the Ministry of Health of the Russian Federation, 10 Zhukov St., Obninsk, 249036, Russia; MD, DSc, Head of the Pathomorphology Department; The A. Tsyb Medical Radiological Research Centre — the Branch of National Medical Research Radiological Centre of the Ministry of Health of the Russian Federation, 10 Zhukov St., Obninsk, 249036, Russia; Head of Histology and Immunohistochemistry Department, Institute of Translational Medicine and Biotechnology; First Moscow State Medical University named after I.M. Sechenov (Sechenov University), 8/2 Trubetskaya St., Moscow, 119991, Russia; PhD, Research Laboratory Assistant, Laboratory of Tissue Engineering; The A. Tsyb Medical Radiological Research Centre — the Branch of National Medical Research Radiological Centre of the Ministry of Health of the Russian Federation, 10 Zhukov St., Obninsk, 249036, Russia; MD, PhD, Head of the Biotechnology Research and Production Complex; The A. Tsyb Medical Radiological Research Centre — the Branch of National Medical Research Radiological Centre of the Ministry of Health of the Russian Federation, 10 Zhukov St., Obninsk, 249036, Russia; Deputy Head of the Center for Collective Use of Scientific Equipment “Radiological and Cellular Technologies”; National Medical Research Radiology Centre of the Ministry of the Russian Federation, 4 Korolev St., Obninsk, 249036, Russia; PhD, Associate Professor; Obninsk Institute for Nuclear Power Engineering — the Branch of National Research Nuclear University MEPHI, 1 Campus Territory, Obninsk, 249034, Russia; Researcher; Peoples’ Friendship University of Russia named after Patrice Lumumba, 6 Miklukho-Maklaya St., Moscow, 117198, Russia; Head of Regenerative Medicine Department; National Medical Research Radiology Centre of the Ministry of the Russian Federation, 4 Korolev St., Obninsk, 249036, Russia; MD, DSc, Corresponding Member of the Russian Academy of Sciences, Director; The A. Tsyb Medical Radiological Research Centre — the Branch of National Medical Research Radiological Centre of the Ministry of Health of the Russian Federation, 10 Zhukov St., Obninsk, 249036, Russia; Professor of the Department of Oncology and X-ray Radiology named after V.P. Kharchenko, Medical Institute; Peoples’ Friendship University of Russia named after Patrice Lumumba, 6 Miklukho-Maklaya St., Moscow, 117198, Russia; MD, PhD, Head of the Center for Innovative Radiological and Regenerative Technologies; National Medical Research Radiology Centre of the Ministry of the Russian Federation, 4 Korolev St., Obninsk, 249036, Russia; MD, DSc, Professor, Academician of the Russian Academy of Sciences, General Director; National Medical Research Radiology Centre of the Ministry of the Russian Federation, 4 Korolev St., Obninsk, 249036, Russia; Head of the Department of Urology and Operative Nephrology with a Course of Oncourology, Medical Institute; Peoples’ Friendship University of Russia named after Patrice Lumumba, 6 Miklukho-Maklaya St., Moscow, 117198, Russia

**Keywords:** tissue engineering, 3D bioprinting, scaffold, bioinks, ADSCs

## Abstract

**The aim of the study** was to analyze the composition feasibility of the bioinks based on high-concentration collagen (40 mg/ml) and gelatin granules (6.25 mg/ml) for bioprinting of porous-structured hydrogel constructs using an extrusion 3D bioprinter.

**Materials and Methods:**

Bioprinting was performed on a 3D Invivo bioprinter (Rokit, South Korea). We assessed the filament continuity during extrusion, the changes in its thickness after test printing and incubation, as well as the biodegradation of prepared scaffolds. The hydrogel cytocompatibility was studied by the proliferation of adipose-derived stem cells (ADSCs) incorporated into the scaffolds. Flow cytometry was performed to determine the immunophenotype of ADSCs. Cell proliferation in the scaffold structure was studied *in vitro* during 28 days spectrophotometrically after adding PrestoBlue reagent. The expression of target genes was analyzed by quantitative reverse transcription polymerase chain reaction (RT-PCR) on day 21 of cultivation. We used the primers for mRNA encoding the synthesis of chondrogenic factors and metabolites (*ACAN*, *SOX9*, *COL1A1*, *COL2A1*), surface markers (*CD29*, *CD44*, *CD73*, *CD90*, *CD105*), as well as hypoxia (*HIF1A*), proliferation (*PCNA*), and apoptosis (*BCL2*, *BAX*) factors. The morphology of the scaffolds was studied on day 28 of culturing by light microscopy after fixing and staining the histological sections.

**Results:**

The extrusion of the high concentration collagen-based hydrogel composition (40 mg/ml) and gelatin granules (6.25 mg/ml) during printing was stable, there was no filament breakage. When incubated in phosphate-buffered saline, the filament thickness of the hydrogel was statistically significantly higher than the scaffold thickness after printing. The degradation of the scaffolds from the hydrogel and gelatin in the solution of type I collagenase started earlier than the collagen scaffolds. The incubation in phosphate-buffered saline for 14 days resulted in less mass loss when drying the collagen scaffolds with gelatin granules. The cells isolated from human adipose tissue expressed surface markers characteristic of ADSCs. ADSCs proliferation and differentiation in chondrogenic direction were observed in both groups compared. The differences were in the spatial arrangement of the cells. In the collagen scaffolds the most cells were on the surface, while in the scaffolds from collagen and gelatin the cells were distributed throughout the whole volume. The 2^–ΔΔCt^ quantitative reverse transcription polymerase chain reaction results showed the increased expression of the transcription factor *SOX9* by the cells in the collagen and gelatin scaffolds, as well as the decreased expression of the anti-apoptotic gene *BCL2* relative to the collagen scaffolds.

**Conclusion:**

The present study suggested the bioink composition based on high concentration collagen (40 mg/ml) and gelatin granules (6.25 mg/ml) for bioprinting of porous-structured hydrogel constructs. The study showed hydrogel to be appropriate for printing and exhibited the properties of a porous material. The hydrogel provided the uniform distribution of ADSCs in the scaffold volume, contributing to their differentiation in the chondrogenic direction. Thus, the suggested bioink composition appears to be a promising material to be used in tissue engineering.

## Introduction

Cartilage damage frequently results from injuries, chronic and autoimmune diseases. Human body has limitations in cartilaginous tissue regeneration due to its avascular structure and a low metabolic activity of chondrocytes [1, 2]. Currently, surgical procedures of cartilage recovery are based on microperforation, chondroplasty, and the transplantation of cadaver materials. However, instead of the complete recovery of cartilage defect continuity, these techniques just remote the recurrence [3, 4]. It prompts continuing the searching for novel methods to develop tissue-engineered analogs of cartilaginous tissue.

Three-dimensional (3D) extrusion bioprinting is an additive manufacturing technology for the layer-bylayer printing of cytocompatible hydrogel materials [5]. The bioprinting process itself involves the creation of a tissue-engineered construct (scaffold). This scaffold is formed by depositing a hydrogel bioink containing encapsulated cells directly onto the bioprinter’s build platform or a culture plate using an automated dispensing system [6]. Hydrogel formulations containing cells and other necessary components (such as culture medium, buffer solutions, and cross-linking agents) used in 3D bioprinting are referred to as bioinks [7]. The development of new biocompatible materials for 3D bioprinting and the optimization of existing compositions are among the primary objectives for both tissue engineering and regenerative medicine as a whole [8].

The studies on cartilage remodeling oftimes use differentiated cells, such as chondrocytes and the cells at tissue-specific differentiation stage — chondroblasts [9]. The procedure involves obtaining cells from healthy cartilaginous tissue and their expansion under *in vitro* conditions. The drawback of the method is the limited availability of chondrocytes and their tendency to undergo dedifferentiation into fibroblast-like cells after 2 weeks of cultivation on adhesive plastic surfaces [10, 11]. Moreover, there can be damaged a donor area in the cartilaginous tissue retrieval spot [12, 13].

Lipoaspirate obtained from adipose tissue containing adipose-derived stem cells (ADSCs) can be an alternative source of cellular material [14]. The major advantage of ADSCs is the less invasive procedure of obtaining in contrast to chondrocytes and chondroblasts; high yield of cells, as well as their ability to chondrogenic differentiation after 6 passage and cryoconservation cycles [15].

The clinical application of full-scale scaffolds with a thickness of 1 mm or more is associated with a number of challenges related both to the bioprinting process itself and the integration of cells into the scaffold [16]. Encapsulated cells are frequently restricted in migration and proliferation by a thick biomaterial network formed during bioink gel-formation.

**The aim of this study** was to evaluate the feasibility of a bioink composition based on high-concentration collagen (40 mg/ml) and gelatin granules (6.25 mg/ ml) for printing porous-structured hydrogel constructs using an extrusion-based 3D bioprinter. We assessed the composition feasibility for printing, the change in filament thickness after test printing and incubation, the biodegradation of the resulted scaffolds, as well as their cytocompatibility and functionality.

## Materials and Methods

### Bioink mixing procedure

Bioinks were prepared in syringes (1 ml; Vogt Medical, Germany) connected via a plastic combi-fix adapter (connection type Luer Lock; Leiko Injekto, China) following the collagen manufacturer’s protocol [17]. To prepare 1 ml of the composition under study, we mixed 500 μl of sterile porcine type I atelocollagen in concentration 80 mg/ ml (Imtek, Russia), 250 μl Tris-HCl (100 mM; PanEco, Russia), 250 μl of cell suspension in the nutritional medium DMEM (Gibco, USA), 6.25 mg of gelatin granules (G1890; type A, gel strength ~300 g Bloom; Sigma-Aldrich, USA). The bioinks of the same composition, though without gelatin granules, were used for printing control scaffolds.

### Gelatin granule diameter determination

Before mixing, we assessed the diameter of the gelatin granules we used. For this purpose, a granule sample was applied on glasses followed by taking photos using an inverted microscope Biomed-3 (BioMed, Russia) and the camera ToupCam UCMOS03100KPA (ToupTek Photonics, China). The images were processed by software ImageJ v. 1.52a. A diameter was calculated according to the formula:

D=2Sπ,

where *D* — granule diameter, *S* — granule section square.

### Main printing parameters

Bioprinting was performed using a bioprinter 3D Invivo (Rokit, South Korea). The materials were extruded through needles 21G (~514 μm), Luer Lock type. Before printing, the printing table was adjusted, and the pressure in a syringe dispenser was equalized. G-code was created in NewCreatorK (v. 1.57.63). Scaffolds were printed in the form of cylinders, 4 mm high and 3.2 mm in diameter. The parameters were the following: layer height — 514 μm, filling percentage — 80, filling type — concentric, the printing speed of the first layer — 2 mm/s, printing speed — 5 mm/s, the wall thickness — 1.028 mm, the first layer thickness percentage — 133, material yield percentage — 100. Skirt parameter was applied, it consisting in plotting a contour around the printed area to exclude breaking down the material at the first layer. The table temperature and the hydrogel temperature in a syringe was maintained as 4°С.

### Assessment of the line thickness and the niche square

To assess the filament thickness of the obtained hydrogel composition we used a test plate with the line 0.5 mm wide and 8 niches with an area of 4 mm^2^ according to the technique given in our previous works [18, 19]. The object height — 600 μm, the printing layer height — 342 μm. Taking into consideration a potential error of the basic layer calibration, the material yield at the first layer was 115%. After printing the plate was embedded in phosphate-buffered saline (PBS) (PanEco, Russia) and incubated for 24 h at 37°C. After that PBS was removed, and the printed objects were photographed using an inverted microscope Biomed-3 (BioMed, Russia) and camera ToupCam UCMOS03100KPA (ToupTek Photonics, China). The images were processed in the program ImageJ v. 1.52a.

### Biodegradation assessment of tissue engineered constructs

To assess biodegradation time 2 groups of scaffolds were printed: 4% collagen scaffolds and 4% collagen with incorporated gelatin granules at the concentration of 6.25 mg/ml. After 3D printing the scaffolds were embedded into the warm PBS solution (37°С) and placed into СО_2_-incubator MCO-5AC (Sanyo, Japan) for 24 h to complete polymerization. Next day the samples were divided into two groups. The biodegradation of the obtained scaffolds was studied in incubation in PBS and type I collagenase solutions at the concentration of 0.1 mg/ml (Gibco, USA). The degradation degree after incubation in the collagenase solution was assessed 2, 4, and 6 h later; in PBS solution — in 7 and 14 days. The samples were kept at 37°С followed by PBS washing twice. The scaffolds (n=5) were put into Petri dishes by groups, and left for drying at room temperature till next day. Then the scaffolds were placed into a drying chamber GP-40 МО (Stock Company “Kasimov Instrument Factor”, Russia). Program “Drying” (85°С) was used for an hour. The scaffolds were weighed using analytical scales Adventurer Pro AV114C (Ohaus, USA).

### Culture of adipose-derived stem cells

Experimental studies on cytocompatibility were carried out using ADSCs. Adipose tissue was donated during planned liposuction surgeries after the patients signed an informed consent. The cells isolated using type II collagenase (Sigma-Aldrich, USA) were brought into culture flasks at the rate of 5·10^4^ cells per 1 cm^2^ and cultivated in DMEM (PanEco, Russia) containing 1 g/L glucose and adding 10% fetal bovine serum — FBS (Biosera, France), penicillin–streptomycin (100 U/ml and 100 μg/ml, respectively), glutamine (150 μm/ml) in СО_2_-incubator MCO-5AC (Sanyo, Japan) at 37°С till the 9^th^ passage. The cell morphology was assessed by phase-contrast light microscopy using an inverted microscope Leica DMi1 (Leica Microsystem, Germany). The cells were imaged using camera Flexacam C1 (Leica Microsystem, Germany). To estimate the cell viability before they were incorporated into scaffolds, the suspension was stained with 0.4% trypan blue solution (PanEco, Russia) in PBS.

### Flow cytometry

Before incorporation into bioink structure, we performed flow cytometry using Macs Quant 16 (Miltenyi Biotec, Germany) to determine the immune phenotype of ADSCs. The cells were removed from the plastic using trypsin solution EDTA (PanEco, Russia) followed by washing the cells free from PBS enzymes (400 g, 5 min) and bringing into test tubes Falcon 12×75 mm (Thermo Fisher Scientific, USA) in amounts of 5·10^5^ cells per 50 μl staining the buffer (PBS with 1% FBS). Then there were added murine monoclonal antibodies to CD29, CD90, СD105 (130101-27, 130-114-859, 130-112-163; Miltenyi Biotec, Germany); CD44, CD73 (REF 347943, REF 561254; Becton Dickinson, USA); CD45, CD34 (SAB4700480, SAB4700682; Sigma-Aldrich, Germany) in accordance with the manufacturer’s instruction. The cells were incubated for 30 min at 4°С in a refrigerator followed by centrifugation and washing in PBS twice, and making the sample volume up to 400 μl. Each sample was reported in three repeats, the number of events being 30,000. To measure FITC fluorescence (CD44, CD73, CD90, CD45) channel B1 was used (525/50 nm), for PE (CD29, СD105, CD34) — channel B2 (579/34 nm). The cells, which were not stained by antibodies, served as the negative control.

### Proliferation assessment of the cells incorporated into the scaffolds

The constructs with ADSCs were printed to study the proliferation of the cells inside the scaffolds (concentration — 2·10^6^ in hydrogel, 1 ml). Two groups of scaffolds were studied: the scaffolds printed from 4% collagen hydrogel, and the scaffolds — from bioinks based on collagen and gelatin. The printed cylindrically shaped scaffolds were incubated in 24- well plates (Nunc, Denmark) in chondrogenic medium consisting of DMEM with GlutaMAX (Gibco, USA), and containing 4.5 g/L glucose with added 5% fetal bovine serum (Biosera, France), 1% penicillin–streptomycin (Gibco, USA), 100 ng/ml fibrinoblast growth factor (PanEco, Russia), 100 ng/ml insulin-like type I growth factor (Sigma-Aldrich, USA), 10 ng/ml transforming growth factor (Sigma-Aldrich, USA), 100 IU insulin (Republical Unitary Enterprise “Belmedpreparaty”, Belarus), 10 μg/ml ascorbic acid (50 mg/ml; OJSC “Dalkhimpharm”, Russia) under standard conditions within 3, 7, 14, 21, and 28 days. The medium was changed every 2 days. After incubation the scaffolds were placed into a 96-well plate, embedded in the nutritional medium without serum (180 μl/well) followed by adding reagent PrestoBlue (Invitrogen, USA), 20 μl per well. The scaffolds were incubated for 2 h at 37°С. Optical density was measured on spectrophotometer INNO-S (LTeK, South Korea) in the wavelength range 500–630 nm with the spacing 10 nm. Total optical density was measured on two wavelengths — 560 nm (maximum absorption) and 600 nm (reference wavelength).

### Histological examination of the obtained scaffolds

The scaffold morphology was checked on day 28 through preparing fixed histological sections and using light microscopy. The scaffolds were being fixed for 24 h in acid Bouin solution containing 1.3% trinitrophenetol (Sigma-Aldrich, Germany) and 40% formalin (BioVitrum, Russia). After washing in 70% ethanol there was the standard histological processing of the samples with their following embedding in paraffin medium Histomix (BioVitrum, Russia). Paraffin sections 5 μm thick prepared on microtome RM2235 (Leica Microsystem, Germany) were placed on silanized glasses S3003 (Dako, USA). For histological examination the paraffin sections were hematoxylin-eosin and alcian blue stained 8GX (Sigma-Aldrich, Germany). After dehydratation in alcohol and clarification in orthoxylene, the preparations were embedded in Canadian balsam (Merck, Germany). Immunohistochemistry was carried out using monoclonal rabbit antibodies to type II collagen (SAB4500366; 1:50; Sigma-Aldrich, Germany). For rabbit antibodies immunoimaging we used the secondary goat antibodies to rabbit IgG conjugated with horse-radish peroxidase (ab205718; 1:1000; Abcam, USA). The solutions for immunohistochemistry were prepared on PBS. According to immunohistochemistry protocol, before applying the primary antibodies to type II collagen, the deparaffinized sections embedded in citrate buffer (рН 6.0) were boiled for 5 min. Endogenic peroxidase was blocked in 3% peroxide of hydrogen. 2% normal serum, 1% bovine serum albumin and 0.1% Triton Х-100 (Sigma-Aldrich, USA) were added into the blocking buffer. The preparations were incubated in the solution of primary antibodies for a night in a wet chamber at 4°С. After washing the preparations in PBS, the sections were applied the secondary goat anti-rabbit antibodies for 1 h at room temperature. Substrate peroxidase was revealed using diaminobenzidine (Liquid DAB+; К3468; Dako, Denmark). After dehydratation in alcohol and clarification in xylene, the preparations were embedded in Canadian balsam. The histological sections were studied using the microscope 3 LUM LED (Micromed, Russia) with an integrated camera U3CMOS05100KPA ToupCam (ToupTek Photonics, China).

### Expression analysis of target genes by quantitative reverse transcription PCR

Total RNA was obtained using reagent ExtractRNA (Eurogene, Russia) from the scaffolds cultivated for 21 days in a chondrogenic medium. For this purpose, the scaffolds were placed into Eppendorf like test tubes (1.5 ml; NEST, China) followed by adding 100 μl of reagent ExtractRNA, then the constructs were pestled with a polypropylene pestle till the homogeneous condition. After that we added ExtractRNA, 1000 μl; the test tube content was stirred on vortex (V-1 plus; BioSan, Latvia) for 16 s. Next stages were carried out according to a standard manufacturer protocol. To increase the purity degree of the isolated RNA, we used precipitation in 100 μl of LiCl solution (12 М; 310468; Sigma-Aldrich, USA). ADSCs culture of the 9^th^ passage cultured in Petri dishes (60 mm; Wuxi NEST Biotechnology Co., Ltd, China) served as the control. RNA concentration was measured on a spectrophotometer Nanodrop ND-1000 (Thermo Fisher Scientific, USA). Complementary DNA was synthesized using a set for reverse transcription M-MuLV–RH (R03-10; Biolabmix, Russia) according to a manufacturer protocol using random hexanucleotide primers on an amplifier BIS М111-05-60 (LLC “BIS-N”, Russia). All samples of the complementary DNA were kept at -20°C prior to quantitative PCR analysis.

Quantitative PCR was performed in a 96-well plate (B96NS-01N; GenFollower, China) using a set BioMaster HS-qPCR SYBR Blue 2× (МНС030-2040; Biolabmix, Russia) on the device CFX96 Real-Time System (BioRad, USA). The sequences of the primers used for quantitative PCR (see the Table) were designed using an online tool Primer-BLAST NCBI [20]. The primers were synthesized in LLC “Bigl” (Russia). Quantitative PCR was performed in three repeats for each sample. Preliminary denaturation — 95°C, 5 min; 40 cycles: denaturation (95°C, 10 s), annealing (61°C, 20 s), elongation (72°C, 10 s). Relative expression levels of the matrix RNA were stated using 2^–ΔCt^ method depending on the reference. In the collagen scaffolds and the scaffolds based on collagen and gelatin, using 2^–ΔΔCt^ method, we determined the fold changes of the expression levels. Reference genes were chosen among five candidate genes: *ALAS1*, *GAPDH*, *GUSB*, *IPO8*, and *YWHAZ* according to the technique by Xie et al. [21].

**Table:** Sequence of the primers used in the experiment

Gene	Primer sequence 5’→3’ (F — forward, R — reverse)	Size of PCR product, base pairs
*ALAS1*	F: TTGGGGATCGGGATGGAGTC R: GAGAACTCGTGCTGGCGATG	102
*GAPDH*	F: CCTCTGACTTCAACAGCGACA R: GTTGTCATACCAGGAAATGAGCTTG	101
*GUSB*	F: GCGTAGGGACAAGAACCACC R: TCCAAGGATTTGGTGTGAGCG	120
*IPO8*	F: GCAGAGTGTCATGCAGCTAAAC R: GACCCCTCGAGTTAATCTCTCCA	120
*YWHAZ*	F: TGGTGATGACAAGAAAGGGATTGT R: AGTTAAGGGCCAGACCCAGT	119
*SOX9*	F: GGCAAGCTCTGGAGACTTCTG R: CCCGTTCTTCACCGACTTCC	138
*ACAN*	F: ACACTGGCGAGCACTGTAAC R: GCTGGGAAGGCATAAGCATGT	113
*COL1A1*	F: CTGACGCACGGCCAAGAG R: TCCACACGTCTCGGTCATGG	106
*COL2A1*	F: AAGGATGGCTGCACGAAACA R: TGTCCATGGGTGCAATGTCAA	106
*PCNA*	F: GGCGCTAGTATTTGAAGCACCA R: CACCAGAAGGCATCTTTACTACACA	131
*HIF1A*	F: CATCCAAGAAGCCCTAACGTGT R: TCGCTTTCTCTGAGCATTCTGC	111
*BAX*	F: CGGGTTGTCGCCCTTTTCTA R: GGAAGTCCAATGTCCAGCCC	106
*BCL2*	F: TGATGGGATCGTTGCCTTATGC R: CAGTCTACTTCCTCTGTGATGTTGT	106
*CD105*	F: TAGCCCTGCGTCCCAAGAC R: GGACGAGGCCTTTGCTTGTG	114
*CD90*	F: TCACAGTGCTCAGAGACAAACTG R: AAATCCGTGGCCTGGAGGA	115
*CD73*	F: CAATGGTGGAGATGGGTTCCAG R: TTGATCCGACCTTCAACTGCTG	135
*CD44*	F: ACCCAGAAGGAACAGTGGTTTG R: TTGGATGGCTGGTATGAGCTG	118
*CD29*	F: ACCAACCGTAGCAAAGGAACAG R: AATGTCTGTGGCTCCCCTGAT	107

### Statistical processing

The findings were imaged and analyzed using statistical software R v. 3.4.1 and GraphPad Prism v. 9.5.0. There were applied one-way ANOVA test along with Tukey method. Student t-test was used to assess the filament thickness and niche area, while the Mann–Whitney U test was used for gene expression analysis. The differences were considered significant if p<0.05.

## Results

### Printability of bioink composition

**Figure 1.**
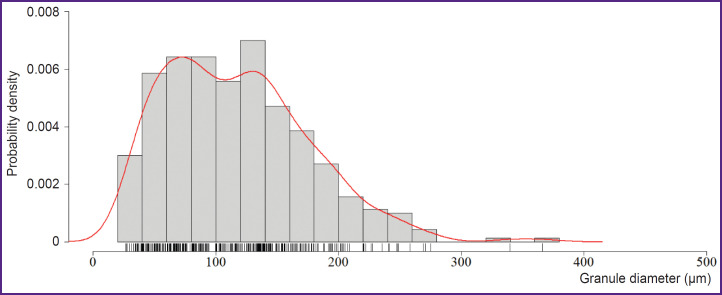
Probability density histogram of the diameter of dry gelatin granules (300 g Bloom) incorporated in the collagen hydrogel (n=350)

The interquartile range of the diameter of gelatin granules incorporated in the collagen hydrogel was [69.26; 154.57] μm, the median being 112.60 μm (n=350) (Figure 1). The hydrogel extrusion through the needles 21G while printing the test plate was stable, there was no filament breakdown. After printing there were revealed no significant differences between the collagen hydrogel and the bioinks based on collagen and gelatin granules (p>0.05).

**Figure 2.**
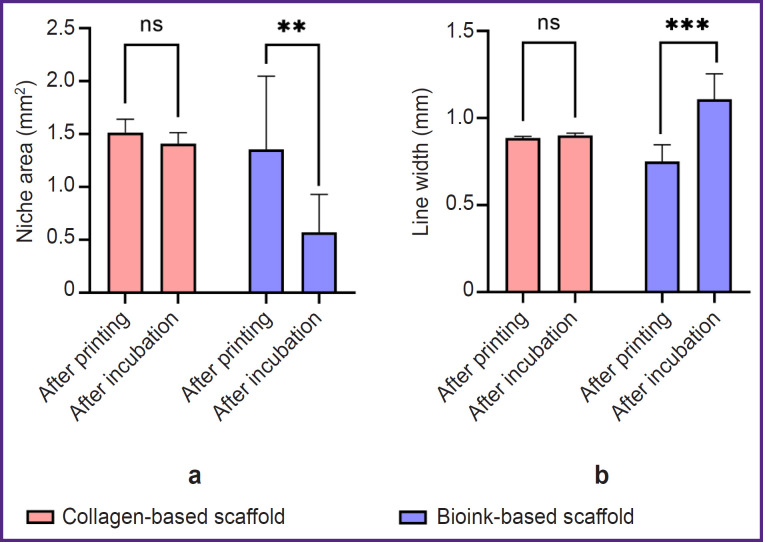
Test printing results of the scaffolds based on 4% collagen and the scaffolds based on bioinks The sizes of niches (а) and lines (b) after printing, and following 24-hour incubation in phosphate buffer saline. The histogram represents mean values and 95% confidential interval; ns — p>0.05, ** p<0.01, *** p<0.001

The incubation of the filament from the hydrogel based on collagen and gelatin in PBS solution resulted in the filament swelling (Figure 2 (b)) leading to both: the filament thickening and the decreased volume of the formed niches (Figure 2 (а)). The swelling degree of the obtained bioink composition for lines increased by 47.4%, for niches — by 42.2%. Thus, the hydrogel from collagen with gelatin granules exhibited the properties of a porous material, the hydratation of which was more pronounced.

### Scaffold biodegradation

**Figure 3.**
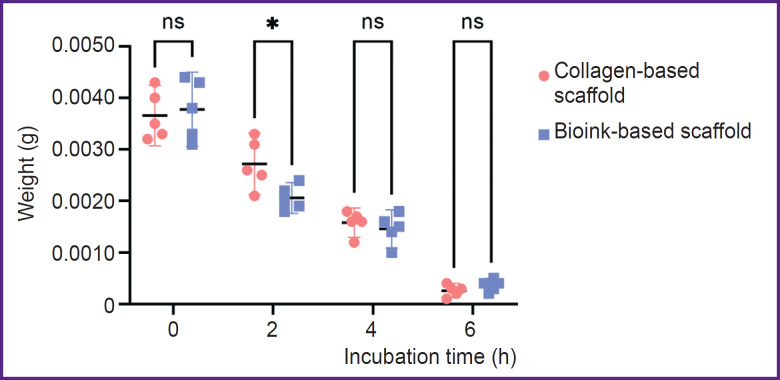
Scaffold weight change when incubated in collagenase solution, at the concentration of 0.1 mg/ml The dot plot shows mean weight values and 95% confidential interval; ns — p>0.05, * p<0.05 (n=5 per each dot)

**Figure 4.**
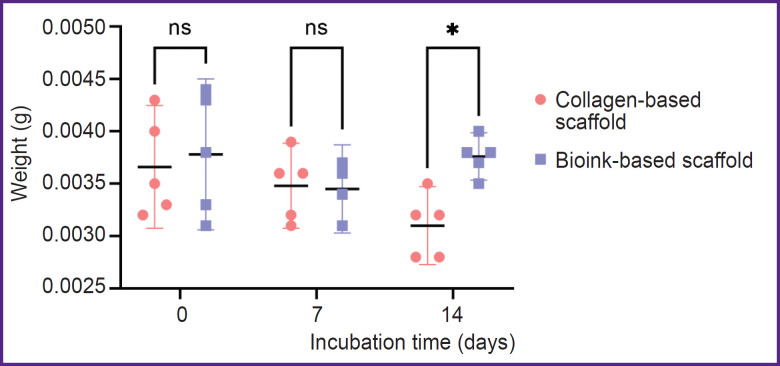
Scaffold weight change when incubated in phosphate-buffered saline The dot plot shows mean weight values and 95% confidential interval; ns — p>0.05, * p<0.05 (n=5 per each dot)

Figure 3 represents the data on the biodegradation of the scaffolds in type I collagenase. There was a statistically significant difference between the groups after 2-hour incubation (p<0.05). The mass of scaffolds from the investigated bioink composition was lower, since the enzymatic degradation of collagen and gelatin molecules began earlier in these scaffolds, likely due to better enzyme accessibility. There were no reliable differences in the weight of the scaffolds of both groups after they were in type I collagenase solution for 4 and 6 h (p>0.05). The dry weight values of the scaffolds incubated in PBS solution (Figure 4) were statistically significantly different between the groups on day 14 (p<0.05). The scaffolds from collagen and gelatin were found to have the higher value. Since the drying time was the same, there might be occurred the change of physical and colloidal properties of the hydrogel when gelatin granules were added to collagen, therefore, its ability to bind water molecules enhanced.

### Culture of adipose-derived stem cells

**Figure 5.**
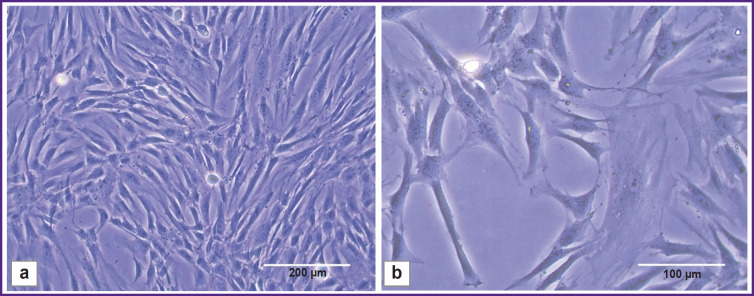
Adipose-derived stem cells Light microscopy; 10× (a); 20× (b)

ADSCs cultivated in a monolayer had fibroblast-like morphology: large spread-eagled fusiform or irregularly shaped cells (Figure 5) with 2–4 long processes; cytoplasm — homogeneous, clear, with no inclusions. The nuclei were located close to periphery (eccentrically) with homogeneous chromatin distribution. The viability of the 9^th^ passage cells was 96.34±0.76%.

### Immunophenotyping of adipose-derived stem cells

**Figure 6.**
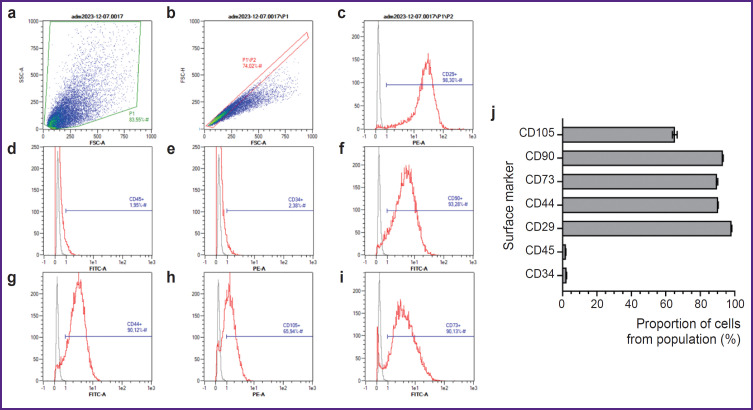
Immunophenotype determination of adipose-derived stem cells (ADSCs) of the 9^th^ passage Density plot of SSC-A vs FSC-A with selected region P1 (a); density plot of FSC-H vs FSC-A with selected region P1/P2 (b); histogram of event count in the PE channel; the P3 region includes events positive for the CD29 (c), CD34 (e), and CD105 (h) clusters of differentiation; histogram of event count in the FITC channel, the P3 region includes events positive for the CD45 (d), CD90 (f), CD44 (g), and CD73 (i) clusters of differentiation. The acquired sample is shown as a red line; the unstained control is shown as a gray line. The percentage of ADSCs positive for the surface markers (j). The bar graph shows mean values and standard deviation

The flow cytometry gating strategy consisted of excluding all cellular debris by plotting a P1 region on an FSC-A vs. SSC-A density plot (Figure 6 (a)). To exclude cell doublets, a P2 region was set using Gate 1 (P1/P2) on an FSC-A vs. FSC-H plot (Figure 6 (b)). The events from Gate 2 were displayed on histograms for PE and FITC fluorescence intensity. The P3 region (P1/P2/P3) identified events with positive expression levels of the surface markers (Figure 6 (c)–(i)). The percentage of cells expressing surface markers characteristic of ADSCs, according to cytometry findings was as follows: 2.35±0.03% for CD34; 1.95±0.02% for CD45; 93.13±0.20% for CD90; 89.73±0.43% for CD73; 90.29±0.17% for CD44; 98.24±0.08% for CD29; 65.22±1.35% for CD105 (Figure 6 (j)).

### Proliferation assessment of the cells inside the obtained scaffolds

**Figure 7.**
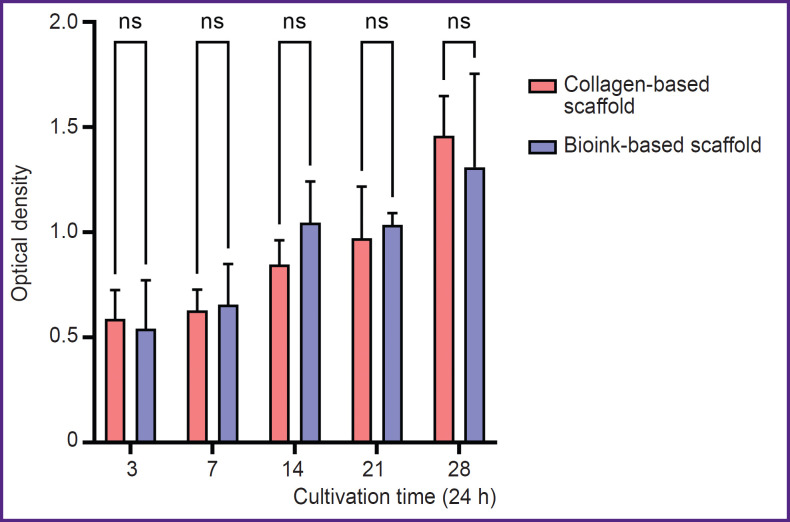
Optical density values after adding reagent PrestoBlue, obtained on days 3, 7, 14, 21, and 28 of incubation of 4% collagen tissue engineered constructs and the scaffold based on bioinks The histogram represents the mean values of optical density and 95% confidential interval; ns — p>0.05

When culturing the scaffolds obtained from collagen hydrogel and the scaffolds from bioinks based on collagen with gelatin granules there was observed the proliferation of incorporated cells during the entire period of observation. There was revealed no significant difference in the mean optical density value between the groups of scaffolds on days 3, 7, 14, 2, and 28 of culturing (Figure 7).

### Histological examination of the obtained scaffolds

**Figure 8.**
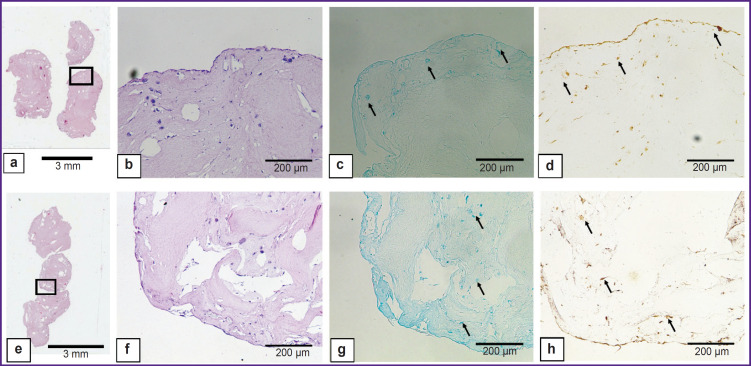
Scaffolds based on collagen hydrogel (a–d) and bioink-based scaffolds (e–h) after 28-day culture Eosin-hematoxylin staining (а, b, e, f), alcian blue staining (c, g), immunohistochemical response to type II collagen (d, h). The arrows indicate the range of interest — the cells synthetizing glycosaminoglycans and type II collagen; 1× (a, e); 10× (b–d, f–h)

The scaffolds after 28-day incubation in standard conditions are represented in Figure 8. ADSCs proliferation and differentiation in chondrogenic direction were observed in both groups compared, and there were found the differences between the groups. In the collagen scaffold the cells were situated solitarily buried in the scaffold and as a uniform layer on the scaffold surface. Type II collagen and glycosaminoglycans were revealed primarily of the collagen scaffold surface; the central part lacked staining. The scaffolds based on collagen and gelatin were observed to have the cells uniting into large groups inside the scaffolds; glycosaminoglycans and type II collagen were distributed throughout the sample, with the most intense staining observed in the cell clusters.

### Expression analysis of target genes

**Figure 9.**
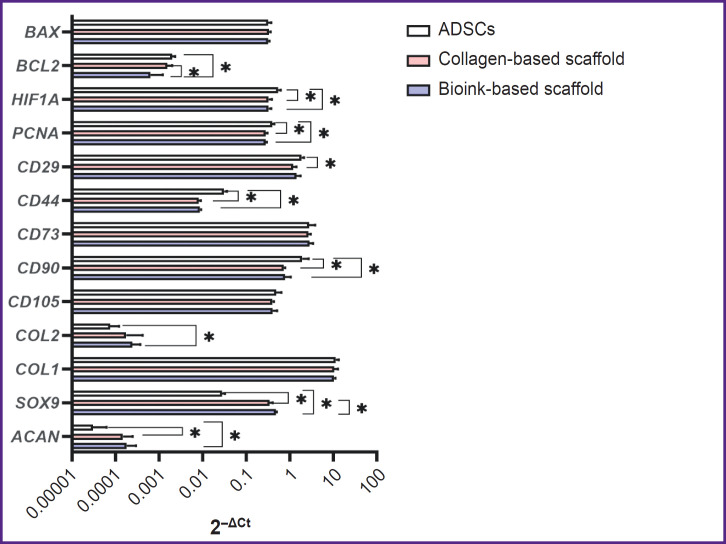
Gene expression analysis using the 2^–ΔCt^ method The histogram represents the mean gene expression values and 95% confidence interval; * p<0,05

**Figure 10.**
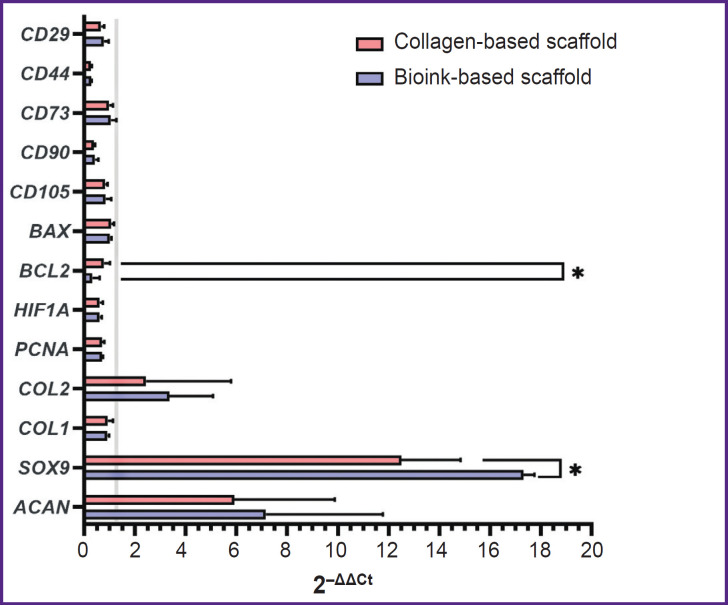
Gene expression analysis by 2^–ΔΔCt^ method The histogram represents the mean values of gene expression and 95% confidential interval; *p<0.05. Grey line — the monolayer culture of adipose-derived stem cells (control)

A pair *GUSB* and *GAPDH* were chosen as reference genes. Using 2^–ΔCt^ method in the scaffolds of two groups under study enabled to observe the differentiation process of ADSCs, which was proved by statistically significant decrease in the gene expression of surface markers *CD44* and *CD90* (Figure 9). *CD90* expression in ADSCs (1.93±0.50) significantly (p<0.05) differed from the expression in the collagen scaffold (0.73±0.04) and the scaffold from collagen and gelatin (0.78±0.17). *CD44* expression in control (0.031±0.0034) significantly differed from the collagen scaffolds (0.008±0.006) and the group of bioink composition under study (0.009±0.0003). Similarly, *CD29* expression in ADSCs (1.85±0.18) significantly (p<0.05) differed from the collagen scaffolds (1.21±0.14). There were no statistically significant differences in the expression of *CD105* and *CD73* markers between three groups. The cells inside the scaffolds from collagen and gelatin granules produced *COL2A1* and *ACAN* genes typical for cartilaginous tissue matrix, as well as the transcription factor gene responsible for chondrogenesis regulation — *SOX9*. The expression of *ACAN* and *SOX9* in two scaffold groups significantly differed from the control. *COL2A1* gene expression by 2^–ΔCt^ method in ADSCs culture (0.00008±0.000028) significantly differed (p<0.05) from the expression level in the scaffolds from collagen with gelatin granules (0.00024±0.000079). There were found no significant differences in *COL2A1* expression in the groups. *PCNA* expression in ADSCs (0.40±0.03) was significantly higher (p<0.05) than in the collagen scaffold (0.28±0.02) and the scaffold from the studied hydrogel (0.28±0.01). *HIF1A* expression in ADSCs (0.54±0.05) significantly differed (p<0.05) from the collagen scaffold expression (0.33±0.04) and in the scaffold from the hydrogel under study (0.33±0.03). The expression of antiapoptotic factor gene *BCL2* for the group of the bioink composition under study (0.0006±0.0004) differed from both — the collagen scaffold (0.0016±0.0003) and the control group (0.0020±0.0002). *BAX* expression showed no significant differences between the groups. Using 2^–ΔΔCt^ method (Figure 10) enabled to reveal statistically significant differences (p<0.05) in *SOX9* gene expression for the collagen scaffolds and the scaffolds from collagen with gelatin granules (12.50±1.47 and 17.32±0.27, respectively). Moreover, there were found the differences in *BCL2* expression (0.78±0.15 and 0.32±0.18, respectively).

## Discussion

The grafts produced using extrusion 3D bioprinting should both — faithfully reproduce the cartilaginous defect form and also provide the proliferation of the cells inside the tissue-engineered construct [22]. The basic limitation in clinical application of scaffolds, which should properly reproduce the real volume of a tissue defect, is the difficulty in delivering nutritional substances, especially oxygen, to cells due to the dense structure of biomaterials. The use of the combination of immiscible phases enables to produce the porous structure providing effective diffusion [23]. Ying et al. [24] used the composition based on gelatin methacryloyl (GelMA) and polyethylene oxide (PEO). The study by Tao et al. [25] had hydrogel GelMa as the main component, and the nanoparticles of β-lactoglobulin (β-LG) in dextran solution served as pore-forming. Both studies observed the uniform cell migration across the whole porous scaffold structure, the increase in their viability and proliferative activity.

The present investigation studied the bioinks based on high-concentration collagen (40 mg/ml) modified by adding gelatin granules (6.25 mg/ml), as the material for bioprinting porous-structured hydrogel constructs. It is recognized that regarding the bioink homogeneity and better printability, microgranules should not exceed 50 μm [26]. The present study involved the granules, [69.26; 154.57] μm in diameter, the median being 112.60 μm that did not prevent the printing process. Bioinks were stably applied by an extrusion attachment layer on layer, with no filament breaking. The hydrogel based on collagen and gelatin exhibited the properties of a porous material. Incubation in PBS following printing had a major effect of changing the geometry of constructs: after incubation the group of scaffolds with gelatin granules added demonstrated the increase in filament volume that complied with the findings of the study by Liu et al. on the porous material based on gelatin [27]. The bioink swelling degree increase for lines was 47.4%, for niches — 42.2%. When being kept in a collagenase solution, the biodegradation of the scaffolds from collagen and gelatin started earlier that implies its porosity. The previous studies [28] showed the porosity of 4% collagen gel with rat chondrocyte concentration 20·10^6^ ml^–1^ was insufficient to provide incorporated cells with oxygen and nutrients. It had a negative effect on their viability within 28 days of cultivation under *in vitro* conditions. The present study used the hydrogel with ADSCs at the concentration of 2·10^6^ ml^–1^, i.e., 10 times less. During *in vitro* cultivation, a dynamic increase in cell proliferation was observed over 28 days in both the scaffolds fabricated solely from collagen hydrogel and those made from the investigated bioink. However, the formation of cell accumulations inside the constructs was found primarily in the scaffolds made from collagen and gelatin granules, while the collagen hydrogel had single cells, and the most cells were on the surface. Thus, the hydrogel porosity increase promoted the improved conditions for incorporated cells.

A number of earlier studies [29–31] indicated the advantage of collagen used as the basic material for scaffolds compared with other materials (GelMA, alginate, agarose, hyaluronic acid, fibrin, decellularized extracellular matrix). Incapsulated cells demonstrated good viability, high DNA content, as well as the increased expression of proliferation gene-markers.

ADSCs are known to be able to differentiate in chondrogenic direction [32, 33]. The study by Ichinose et al. [34] described the differentiation process of ADSCs in spheric constructs, where on day 7, in the surface area fibroblasts appeared, and in the middle area — apoptotic cells, in the deep area — chondrocyte-like cells. On day 21 of cultivation there was observed the gradual surface thinning, and chondrocyte-like cells prevailed in cartilage lacunas. The study by Farrell et al. [35] demonstrated the effect of normal oxygen conditions (~21% O_2_) and hypoxia (2% O_2_), as well as glucose (in concentrations 1.0 and 4.5 g/L) on the viability and chondrogenic differentiation of cells in scaffolds. The authors showed glucose deficiency and hypoxia to impair the processes; moreover, glucose deficiency significantly decreased the cell viability and prevented the functional maturation of 3D constructs.

In our study, the scaffolds were incubated in a culture medium with a high glucose concentration. The necessary condition for differentiating stem cells in chondrogenic direction is adding TGF-β3 (transforming growth factor beta 3), BMP2 (bone morphogenetic protein 2) and dextran, along with maintaining their concentrations by nutritional medium renewal [36]. Vitamin С increases ADSCs proliferation, induces chondrogenic differentiation, and enhances a paracrine effect [37, 38]. After *in vivo* scaffold implanting, the hyaline cartilage structure can form due to intensive mass transfer of metabolites [39] and the presence of functionally active macrophages able to merge and form multinucleated cells [40]. A scaffold incorporated in a cartilaginous defect stimulates the migration of the patient’s stem cells and chondrocyte precursors [3, 41].

The comparison of gene expression in cells in the monolayer condition and when being cultivated in scaffold structure helps answering the questions on chondrogenic differentiation time of ADSCs in a scaffold, the changes in phenotype, as well as in division and hypoxia inside constructs. *HIF1A* gene expression emphasizes the hypoxic nature of ADSCs in a monolayer compared to the cells inside a scaffold that complies with the work by Dionigi [42]. *PCNA* expression can be related to both: high division potential of ADSCs and achieving the plateau of the cells in scaffolds on day 21 [43–45].

In the present study, ADSCs *COL2A1* gene expression in the scaffolds based on the studied bioinks were significantly higher compared to the gene expression in the cells cultivated in a monolayer. Type II collagen is a typical marker of chondrocyte differentiation, while type I collagen expression increases during dedifferentiation [46, 47].

Adhesion to plastic, fibroblast-like structure, and the expression of surface receptors of CD105, CD90, CD73, CD44, CD29 are the conditions for typing ADSCs [48]. In the present study, the cells exhibited an immunophenotype consistent with ADSCs: CD105 greater than 65%; CD90, CD73, CD44, CD29 greater than 90%; and CD34 and CD45 less than 3%. CD29 representing the protein integrin β-1 was studied regarding its correlation with the cell ability to differentiate in chondrogenic direction, and it was confirmed by the study by Cicione et al. [49], where the authors emphasized the importance of high CD29 expression level when choosing ADSCs with high chondrogenic potential. It should be noted that CD90 (Thy1) participates in the interaction between cells and extracellular matrix, and an increased CD90 expression indicates the cell ability to differentiate [50]. The study findings of Chang et al. [51] demonstrated antigens CD29, CD44, CD73 and CD90 to express in more than 60% ADSCs at the stage they were isolated. Moreover, the authors concluded the significance of surface markers CD105 and CD166, and they related it to their chondrogenic potential. The expression level of CD105 in the study was over 65% that can suggest the presence of a great deal of chondrogenic precursors among cultivated ADSCs after the 9^th^ passage.

Gene *SOX9* is a key transcription factor regulating chondrogenesis, and acts both — independently and when paired with *SOX5* and *SOX6* forming a chondrogenic trio [52]. It participates in the expression activation of such genes as *COL2A1* and *ACAN*, which are the main macromolecular components of cartilaginous tissue playing a key role in maintaining the normal structure and function of the articular cartilage [53, 54]. ADSCs cultivation in the scaffolds printed from the hydrogel based on collagen and gelatin granules in the chondrogenic medium for 21 days resulted in an increasing expression of *SOX9* gene by the cells. It indicates an active process of their differentiation in chondrogenic direction. The data were consistent with the study by Tao et al. [25], where compared to a standard group, the use of the printed porous hydrogel resulted in the significant increase in *SOX9* expression in the incapsulated cells.

Proteins BCL2 and BAX play a key role in regulating the permeability of mitochondrial membranes and cytochrome C release [55]. In the present study, *BCL2* gene expression in the group of the studied bioinks was significantly lower compared with the collagen scaffolds and the controls. However, there were no significant differences between the groups in *BAX* expression. This may indicate the initiation of the apoptosis mechanism as a result of the chondrogenic differentiation of AD SCs. The findings comply with the study by Wang et al. [56]. The authors indicated the decreased level of *BCL2* expression in the directed neurogenic differentiation of ADSCs following 8-hour induction. The work by Yuan et al. [57] showed the decrease in *BCL2* expression on day 21 after triggering the directed ADSCs differentiation into astrocytes.

## Conclusion

The present study suggested the bioink composition based on high concentration collagen (40 mg/ml) and gelatin granules (6.25 mg/ml) as the material for bioprinting of porous-structured hydrogel constructs. The investigation showed the hydrogel to be appropriate for printing and exhibited the properties of a porous material. The hydrogel provided the uniform distribution of ADSCs in the scaffold volume contributing to their differentiation in the chondrogenic direction. Thus, the suggested bioink composition appears to be a promising material to be used in tissue engineering.
